# Immature Circulating SP-B, Bound to HDL, Represents an Early Sign of Smoke-Induced Pathophysiological Alterations

**DOI:** 10.3390/biom11040551

**Published:** 2021-04-09

**Authors:** Cristina Banfi, Maura Brioschi, Massimo Mapelli, Erica Gianazza, Alice Mallia, Beatrice Zoanni, Elisabetta Salvioni, Paola Gugliandolo, Nicolò Capra, Fabrizio Veglia, Piergiuseppe Agostoni

**Affiliations:** 1 Centro Cardiologico Monzino, IRCCS, 20138 Milano, Italy; maura.brioschi@ccfm.it (M.B.); Massimo.mapelli@ccfm.it (M.M.); erica.gianazza@ccfm.it (E.G.); alice.mallia@ccfm.it (A.M.); beatrice.zoanni@ccfm.it (B.Z.); elisabetta.salvioni@ccfm.it (E.S.); paola.gugliandolo@ccfm.it (P.G.); nicolo.capra@ccfm.it (N.C.); fabrizio.veglia@ccfm.it (F.V.); piergiuseppe.agostoni@ccfm.it (P.A.); 2 Dipartimento di Scienze Cliniche e di Comunità, Sezione Cardiovascolare, Università di Milano, 20122 Milano, Italy

**Keywords:** surfactant protein B, smoke, oxidative stress, inflammation

## Abstract

Cigarette smoking is a major independent risk factor for cardiovascular diseases (CVD). The underlying mechanisms, however, are not clearly understood. Lungs are the primary route of exposure to smoke, with pulmonary cells and surfactant being the first structures directly exposed, resulting in the leakage of the immature proteoform of surfactant protein B (proSP-B). Herein, we evaluated whether proSP-B joined the cargo of high-density lipoprotein (HDL) proteins in healthy young subjects (*n* = 106) without any CVD risk factor other than smoking, and if HDL-associated proSP-B (HDL-SPB) correlated with pulmonary function parameters, systemic inflammation, and oxidative stress. At univariable analysis, HDL-SPB resulted significantly higher in smokers (2.2-fold, *p* < 0.001) than in non-smokers. No significant differences have been detected between smokers and non-smokers for inflammation, oxidation variables, and alveolar-capillary diffusion markers. In a multivariable model, HDL-SPB was independently associated with smoking. In conclusion, HDL-SPB is not only a precocious and sensitive index of the acute effects of smoke, but it might be also a potential causal factor in the onset of the vascular damage induced by modified HDL. These findings contribute to the emerging concept that the quality of the HDL proteome, rather than the quantity of particles, plays a central role in CVD risk protection.

## 1. Introduction

Smoking is firmly established as one of the main cardiovascular risk factors [1]. However, the mechanisms by which cigarette smoking increases the risk of cardiovascular diseases (CVD) are not understood [2]. Accumulating experimental and clinical data have indicated that increased oxidative stress, inflammation, endothelial dysfunction, platelet activation, as well as alterations in the lipid profile have been considered as the potential mechanisms of cigarette smoking increasing CVD [3]. Further, alterations in the enzymes that control lipid transport may be a key underlying mechanism contributing to the smoking-induced high-density lipoprotein (HDL) dysfunctionality [4,5]. Indeed, HDL is susceptible to oxidative modifications by cigarette smoking, so that HDL becomes dysfunctional and may lose its atheroprotective properties in smokers. However, it is unclear how smoke triggers all of the above mechanisms.

The primary entry pathway of cigarette smoke are clearly the lungs, and mainly the alveolar-capillary membrane, which has a relevant surface area and is the barrier between air and circulation. Indeed, tobacco smoke exposure induces alveolar inflammation, which results in an unphysiological release into the bloodstream instead of the alveolar space of surfactant protein B (SP-B), a protein vital for normal lung function [6,7]. Previous observations linked plasmatic SP-B, measured by assays that do not discriminate between immature or mature proteoforms, to the smoking status. Robin et al. [7] found for the first time that serum SP-B was strongly correlated with tobacco smoke exposure in subjects over 50 years old with a high index of cumulative smoking. Moreover, higher levels of circulating SP-B independently associate with aortic atherosclerosis and correlate with a dose–response effect with smoking habit in a population of 30–65 aged subjects with traditional cardiovascular risk factors beyond smoking, suggesting that SP-B may be a useful marker of the dose-dependent vascular effects of smoking [8]. Further, circulating immature proteoform of surfactant protein B (proSP-B) has been proposed as the most reliable lung-specific circulating marker for alveolar-capillary membrane dysfunction and overall clinical status of heart failure (HF) [9]. We have previously demonstrated that circulating proSP-B might rise to the role of theranostic biomarker due to its capability of functioning as a diagnostic and therapeutic biomarker [6,9,10]. We also showed that circulating proSP-B is not present in a free form, but it mainly binds to isolated HDL, and it impairs the anti-oxidant properties of HDL [11], shedding light on proSP-B as a molecule that contributes to the reduction of the defense against oxidative stress, a key mediator in the pathogenesis of HF [12].

Of note, large cohort studies showed a strong inverse relationship between HDL-cholesterol (HDL-C) levels and the risk of incident atherosclerotic CVD [13]. However, the causal relationship between HDL-C and CVD and its usefulness as a drug target for decreasing CVD risk have been disputed by the results of genetic studies as well as by pharmacological intervention trials [14]. These considerations have contributed to the emerging concept that the quality, rather than the quantity, of HDL plays a central role in CVD risk protection [15,16,17,18]. In this regard, HDL quality is evolving as a possible diagnostic marker for cardiovascular outcome in HF [19], and the protein composition of HDL plays a key role in mediating its cardioprotective functions [15].

Of note, all these data have been obtained in subjects with CVD or relevant risk factors. However, the beginning of the story, i.e., whether there is a link between smoke, healthy lung, proSP-B release in the bloodstream, and abnormal HDL, is still unknown.

## 2. Materials and Methods

### 2.1. Patients and Control Subjects’ Characteristics

A cohort of healthy subjects equally distributed in terms of age, gender, and cigarette smoking habits was recruited at Centro Cardiologico Monzino in 2018–2019. The study was approved by the Ethics of Istituto Europeo di Oncologia and Centro Cardiologico Monzino (registration number R853/18-CCM897), and it complies with the Declaration of Helsinki. All participants gave their informed consent before taking part. The inclusion criteria were age below 40 years, the absence of risk factors for CVDs including overweight (BMI > 25.0 kg/m^2^), hypertension (systolic blood pressure >140 +/− diastolic blood pressure >85 mmHg at rest or antihypertensive medication), hyper/dyslipidemia (anamnestic statin therapy), diabetes mellitus (HbA1c > 6.5 rel%). All subjects underwent blood sampling and standard pulmonary function tests. Demographical and clinical data were collected at enrolment. All subjects completed a questionnaire concerning smoking habits. From the venous blood, lipid profile and complete blood count, including white blood cells (WBC) count were measured by standard biochemical procedures, while high-sensitivity C Reactive Protein (CRP) were measured by immunoturbidimetry, and interleukin-6 (IL-6) by enzyme-linked immunosorbent assays (R&D Systems, Minneapolis, MN, USA). Smoking habits are expressed as the number of daily smoked cigarettes, smoking years, or pack-years, which is calculated according to the formula: (number of daily smoked cigarettes per day/20) × number of years of smoking.

### 2.2. Pulmonary Function Tests with Lung Diffusion Measurements

Standard pulmonary tests were performed according to the American Thoracic Society criteria [20]. Lung diffusion for carbon monoxide (DLCO) and nitric oxide (DLNO) were simultaneously measured in the standard sitting position through the single-breath technique, with a breath-hold time of 4 s (MS-PFT analyzer, Jaeger Masterscreen, Hoechberg, Germany).

### 2.3. Assessment of proSP-B

HDL were isolated by sequential ultracentrifugation [21], and proSP-B levels were assayed by immunoblotting as previously described [9]. Briefly, equal amounts of HDL proteins were loaded on 15% polyacrylamide gels using a tris-tricine buffer system in non-reducing conditions, and pro-SP-B was detected by immunoblotting using a primary antibody against SPB (mouse anti-human SPB F-2, Santa Cruz Biotechnology) and quantified by densitometric analysis with QuantityOne software (version 4.5.2, Bio-Rad laboratories, Milan, Italy). In our experimental conditions, the proteoforms of SP-B (~42, 24 and 17–21 kDa) are ascribed to the immature pro-SPB and not to its mature form, as they are detectable also in reducing conditions. For each subject, the values were normalized versus the band volume of a pooled sample, loaded as a control on each gel, and were expressed as arbitrary units (AU) as described [9].

### 2.4. Quantitation of Cysteinylated Albumin by Mass Spectrometry

The relative composition of albumin isoforms was evaluated, as previously described [22], by direct infusion using the Xevo TQ-S micro triple quadrupole mass spectrometer coupled with the M-Class UPLC system (Waters Corporation, Milford, MA, USA). Briefly, plasma samples centrifuged at 3000× *g* for 10 min at 4 °C were diluted 500-fold in 50% acetonitrile containing 0.1% formic acid. After centrifugation at 14,000× *g* for 10 min at 4 °C, 2 µL were injected at 5 µL/min and the spectra were acquired for 6 min with the following parameters: Positive ESI mode; mass range, 1100–1350 m/z; capillary voltage, 3 kV; cone, 90 V; desolvation temperature, 350 °C; source temperature, 150 °C. Data processing for deconvolution was performed with the MaxEnt1 function on the Masslynx software (Waters Corporation, Milford, MA, USA). Mercaptoalbumin (HSA-SH) and cysteinylated albumin (HSA-cys, +120 ± 2 Da) were detected and their intensities were used to calculate the relative abundances as previously described [22].

### 2.5. Statistical Analysis

Being mostly not normally distributed (Kolmogorov–Smirnov test), quantitative variables are reported as median and inter-quartile range unless otherwise stated; categorical variables are shown as count and percentage. Differences between smokers and no-smokers are tested by non-parametric test (Wilcoxon signed-ranked test) for quantitative variables and by chi-square test for categorical variables. Univariable associations between smoking habits, lipids, inflammation, and oxidation parameters and measures of lung function were analyzed by Spearman correlation. General linear models were employed to investigate the association between HDL-associated proSP-B (HDL-SPB) levels and three different measures of smoking intensity: Cigarettes/day, smoke duration (years), and pack-years. Two models with different levels of adjustment for potential confounders were employed: Model 1, adjusted for age, gender, and HDL-cholesterol (HDL-C) levels; Model 2 adjusted for variables in model 1 plus inflammation variables (IL-6, CRP and WBC) and oxidation variables (HSA-cys). The strength of the associations between potential predictors and HDL-SPB was quantified by the partial R^2^. Variables with approximately log-normal distributions were log-transformed before analysis. Statistical analyses were carried out with the SAS statistical package v. 9.4 (SAS Institute Inc., Cary, NC, USA). All tests were 2-sided, and *p* values <0.05 were considered statistically significant.

## 3. Results

### 3.1. Effects of Smoking on Pulmonary Function Tests

A total of 106 healthy subjects were evaluated: 56 females (age: 30 (27–32.5), median (IQR); 26/56 active smokers) and 50 males (age: 32 (28–34); 24/50 active smokers). Among smokers, the median of the number of daily cigarettes, reflecting current smoking, was 7 (5–10, IQR) and 10 (5–15, IQR), for females and males, respectively (*p* = 0.076). Regarding cumulative smoking, pack-years were 3.8 (1.75–6.78, IQR) and 5 (3.6–7.5, IQR) for females and males, respectively (*p* = 0.176); years of smoking were 10 (6–16.5, IQR) and 10 (10–15, IQR) for females and males, respectively (*p* = 0.5416). Main laboratory and clinical characteristics of the enrolled population are summarized in Table 1.

The present population of young adults showed normal resting spirometry and alveolar-capillary diffusion considering both carbon monoxide (DLCO) and nitric oxide (DLNO) as diffusion markers (Table 1). Similarly, no correlation between DLCO or DLNO and HDL-SPB levels was found in the univariable analysis (Table 1).

### 3.2. HDL-Associated ProSP-B Levels Are Associated with Smoking Habits

**Figure 1:**
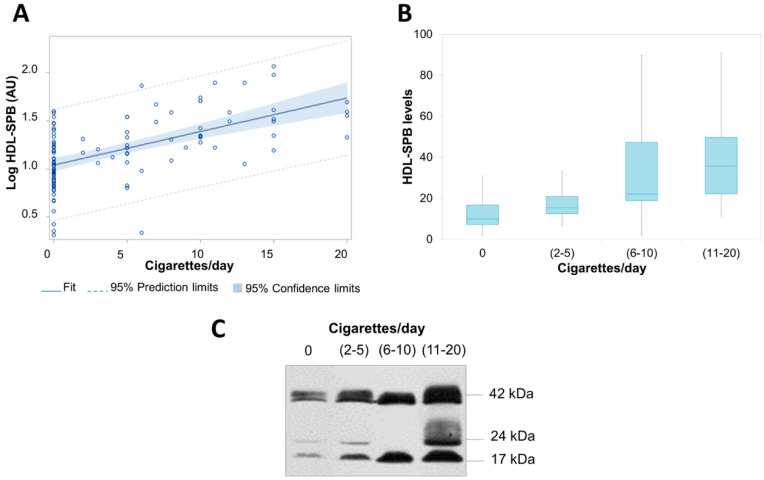
Levels of high-density lipoprotein-associated immature surfactant protein B (HDL-SPB) and current smoking. (**A**) Association of log-transformed HDL-SPB and number of daily smoked cigarettes (cigarettes/day). *p* < 0.0001 by linear regression. (**B**) Distribution of HDL-SPB in relation to tertiles of daily smoked cigarettes. (**C**) Representative image of the HDL-SPB analysis obtained by immunoblotting technique in a no smoker and in smokers from each tertile of daily smoked cigarettes. *p*-value for trend was assessed by Spearman correlation; *p* < 0.0001.

HDL-associated immature proSP-B (HDL-SPB) was measured in all subjects. At univariable analysis, HDL-SPB resulted in being significantly higher in smokers (2.2-fold, *p* < 0.0001, AU 22 (15–38.9)) than in non-smokers (AU 9.9 (7.2–16.7)) (Figure 1). As expected, a strong correlation was evidenced between HDL-SPB and pack-years, smoking years, or the number of daily smoked cigarettes (Table S1, Figure 1).

Moreover, daily smoked cigarettes and HDL-SPB levels negatively correlate with HDL-C (Spearman correlation, r = −0.238, *p* = 0.029, for the number of daily smoked cigarettes and r = −0.351, *p* = 0.0011 for HDL-SPB) (Table S1).

Notably, in a multivariable model, adjusting for age, gender, and HDL-C, HDL-SPB levels remain strongly associated with the number of daily smoked cigarettes, pack-years, and years of smoking (Table 2). Among the associations between HDL-SPB levels and the three smoking variables, pack-years, and number of daily smoked cigarettes have the same magnitude, while years of smoking is the weakest (partial R^2^ = 25.82% for pack-years versus 25.78% for cigarettes/day and 17.85% for smoking years).

### 3.3. Association of HDL-SPB Levels and Smoking Is Independent of Inflammation and Oxidative Stress

In order to assess the presence of an inflammatory state, two known markers induced by smoke (IL-6 and CRP) [23,24], and WBC count have been analyzed, together with plasma cysteinylated albumin (HSA-cys), a recently recognized marker of oxidative stress [25]. No significant differences have been detected between smokers and non-smokers in the current study population for these variables (Table 1) and, accordingly, no correlation has been found between IL-6, CRP, HSA-cys, or WBC and current or cumulative smoking (Table S1).

Further, the association between HDL-SPB levels and current or cumulative smoking remained highly significant in a general linear model (GLM) including inflammatory and oxidative markers, with an estimated increase of 8% of HDL-SPB for every smoked cigarette. Thus, HDL-SPB was related to smoking independently of HDL-C levels, inflammatory, and oxidative markers (Table 3).

## 4. Discussion

In this study, we showed that the levels of proSP-B bound to HDL increase in relation to current and cumulative smoking in a young population of healthy light smokers (<20 pack-years) with a preserved respiratory function, and with no signs of alveolar-capillary membrane dysfunction. Of note, the increase of HDL-SPB occurs in the absence of any smoke-induced inflammatory or oxidative stress state, which usually occurs in subjects with more prolonged or intense smoking habits [23,24,26]. That said, our findings individuate the first step of smoke-induced cardiovascular system damage.

Pro-SPB has a pivotal role in the complex clinical scenario of HF, where it is increased, it correlates with alveolar-capillary membrane dysfunction [9], and it has a definite prognostic value [10]. Moreover, proSP-B was found to be mainly associated to HDL, and, at higher levels, in HF patients-derived HDL [11]. Further, we demonstrated that proSP-B binding to human HDL particles impairs their antioxidant capacity [11], thus likely contributing to the increased oxidative stress, a well-known mediator in the pathogenesis of HF [12]. Finally, Cardner et al. also found SP-B as the most significantly enriched protein of HDL of patients with coronary heart disease [27].

In the last years, the hypothesis that not the quantity of HDL, but rather its functions and composition, may be more relevant for protection in CVDs [16] has been advanced based on the failure of the HDL-cholesterol-raising therapies to reduce the impact of coronary artery disease. On this topic, recent advancements in proteomic technology have dramatically increased our understanding of the HDL protein cargo. In addition to proteins with well-established functions in lipid transport, iron transport proteins, members of the complement pathway, as well as proteins involved in immune function and acute phase response have been repeatedly identified on HDL particles. In a pathological state, various protein and lipid components of the HDL can undergo alterations, which drive a shift towards a dysfunctional state of the lipoprotein, which becomes pro-oxidant, pro-inflammatory, and lastly pro-atherogenic [18,28]. Among the proteins with multiple important functions that reflect, among others, atherogenesis, inflammation, and oxidative stress [19], we suggest that proSP-B can contribute to the dysfunctional state of HDL.

Smoking is a well-known factor increasing the risk of CVD, but the biological mechanisms linking smoking and atherosclerosis are complex, and they likely include, among others, inflammation, vascular endothelial dysfunction, and lipid abnormalities [3,29]. Moreover, all these data have been obtained using material from cases with established cardio-respiratory diseases.

**Figure 2:**
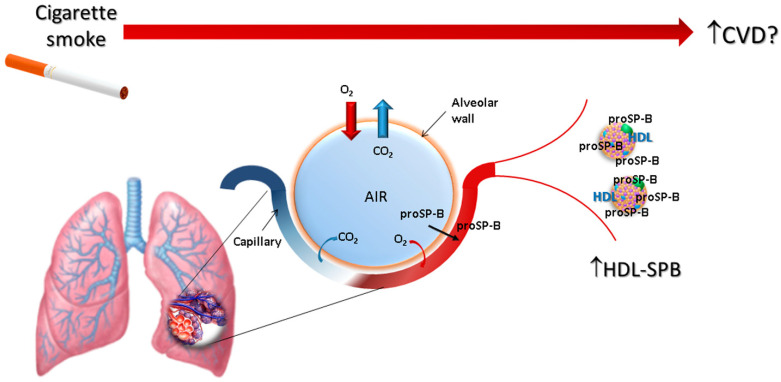
Schematic representation of the hypothesized process linking smoking with an increase of cardiovascular risk, involving the association of proSP-B with circulating HDL.

Our research aims at evaluating the beginning of the cigarette smoke-induced pathophysiological cascade in humans, and this is a unique study in this context, performed in young light smokers without any clinical and functional evidence of lung dysfunction. The results demonstrate that levels of HDL-SPB are strongly correlated with smoking, expressed as pack-year, year of smoking, or the number of daily smoked cigarettes, even in the absence of clinical signs of respiratory functions alteration, CVD risk factors, or inflammatory activation. Notably, the number of daily smoked cigarettes, an index of current smoking, has a stronger correlation with the increase of HDL-SPB than smoking years, suggesting that the increase of HDL-SPB could be the result of an acute smoke-induced insult. Accordingly, we can hypothesize that the first effect of smoke is exerted at the alveolar-capillary pulmonary interface, results in the leakage of immature SP-B, and sees in the abnormal HDL-SPB a key element in the initiation of atherogenesis (Figure 2).

To properly locate our working hypothesis in the context of smoke-associated CVD and to further analyze whether HDL-SPB changes are the first abnormality in smokers, few other findings must be underlined. First, we observed a correlation of lipid levels, and in particular HDL-C, with smoking habits. This is a confirmatory finding since it is well known that HDL and ApoA-I levels are decreased in smokers in a dose-dependent manner [30,31]. Further, cigarette smoking not only alters HDL-C levels but also functions, including the antioxidant and the anti-inflammatory potential, and the ability to promote cholesterol efflux from vascular macrophages [4]. Anyway, in our study, the relationship between HDL-SPB and smoking is preserved independently from HDL-C level.

Moreover, cigarette smoking is known to also affect systemic inflammation, an essential element in the onset and progression of CVDs [32], by activating and releasing inflammatory cells into the circulation and increasing circulating inflammatory mediators such as acute-phase proteins and pro-inflammatory cytokines [23,24,26]. Interestingly, in our population of young, healthy light smokers, we did not find any increase in inflammatory parameters, thus indicating that cigarette smoke may not have influenced yet the inflammatory system in our population, although HDL-SPB is already increased.

Finally, it is well known that the oxidants present in cigarette smoke, as well as oxidants and free radicals endogenously produced by the cellular redox system perturbed by smoke, cause a pro-oxidative environment, likely contributing to accelerate atherosclerosis (discussed in [26]). Therefore, we also assessed the impact of cigarette smoking on the plasma levels of cysteinylated albumin, a recently proposed marker of oxidative stress [25]. However, despite these premises, the levels of cysteinylated albumin were not different between smoker and non-smokers in the analyzed cohort, thus indicating that the perturbations induced by oxidative stress are not yet perceptible in this study population of young light smokers (<20 pack-years, <20 cigarettes/day), and are therefore a later consequence of smoke. Although no signs of inflammatory or oxidative stress, nor functional signs of lung dysfunction were observed in the cohort of patients analyzed, increased amount of proSP-B released into the bloodstream could be an indicator of pulmonary surfactant changes that could be compensated by physiological mechanisms, suggesting the onset of an inflammatory situation in the lung.

A few study limitations need to be acknowledged. First, we do not know how proSP-B binds to HDL; therefore, there is a lack of knowledge regarding the physical binding of proSP-B to HDL. Second, the pathophysiological role of HDL-SPB remains unknown. Indeed, any causal relationship between the alterations in the structural composition and functions of HDL, and the onset of vascular damages, as well as their directionality, needs to be investigated in targeted experiments. However, the binding of proSP-B to HDL is detectable before any clinical and functional sign of lung dysfunction as well as any sign of systemic inflammation and oxidation, suggesting but not proving a causal role of proSP-B release from the alveolar cells in HDL dysfunctionalities.

## 5. Conclusions

In conclusion, we propose a new pathway to explain the deleterious effects of smoke on the cardiovascular system. Indeed, HDL-SPB is both a precocious and sensitive index of the acute effects of smoke, independent of the presence of oxidative and inflammatory states that could appear later, but also a potential causal factor in the onset of vascular damage induced by modified HDL. Accordingly, smoke acts on the alveolar cells by altering SP-B metabolism, so that immature SP-B molecules flow into the bloodstream, where they bind to HDL, modifying its functions. Therefore, impaired alveolar cell SP-B metabolism is likely the first trigger of the smoke-induced pro-atherosclerotic cascade.

## Figures and Tables

**Table 1:** Main clinical characteristics and laboratory data for the study population.

Variable	No Smoker *n* = 56	Smoker *n* = 50	*p*-Value
Age (years)	30(27; 32.5)	32(28; 34)	0.184
Gender (males, *n* (%))	32(57.14%)	26(52%)	0.347
BMI (kg/m^2^)	22.6(20.6; 25)	23(21; 25)	0.357
**Smoking habits**			
smoked cigarettes (*n*)	-	9.5(5; 13)	-
smoking years (*n*)	-	10(6; 15)	-
Pack-years	-	4(2.5; 7.5)	-
**Blood counts**			
HCT %	40.4(36.6; 43.1)	41(38.9; 44.3)	0.149
Hb (g/dL)	14(12.4; 15.3)	14.1(13.5; 15.6)	0.161
PLT (10^3^/μL)	239.5(200.5; 278.5)	226(204; 254)	0.575
RBC (10^3^/μL)	4.7(4.5; 5.1)	4.6(4.4; 5.1)	0.773
WBC (10^3^/μL)	6.3(5.7; 7.3)	6.8(5.8; 8.2)	0.236
MPV (fL)	10.3(10; 10.9)	10.5(9.8; 10.9)	0.779
**Lipids**			
Total cholesterol (mg/dL)	169(155; 187)	167(144; 191)	0.972
HDL cholesterol (mg/dL)	58(48; 67)	51(41; 61)	0.088
LDL cholesterol (mg/dL)	92(79; 111)	94(82; 110)	0.834
TG (mg/dL)	74(59; 97)	86(60; 129)	0.324
**Laboratory data**			
HDL-SPB (AU)	9.9(7.2; 16.7)	22(15.6; 38.9)	<001
CRP (mg/L)	0.7(0.2; 1.8)	0.8(0.3; 1.9)	0.671
IL-6 (pg/mL)	0.87(0.57; 1.16)	0.86(0.56; 1.41)	0.572
HSA-cys (%)	11.03(10.05; 13.62)	10.48(9.5; 11.9)	0.071
**Pulmonary function data**			
DLCO (mL/mmHg/min)	28.2(24.6; 34.6)	27.8(25; 31.2)	0.301
DLNO (mL/mmHg/min)	115.6(99; 141.2)	121.5(101.7; 132.3)	0.982

BMI, body mass index; HCT, hematocrit; Hb, hemoglobin; PLT, platelets; RBC, red blood cells; WBC, white blood cells; MPV, mean platelet volume; HDL, high density lipoproteins; LDL, low density lipoproteins; HDL-SPB, HDL-associated immature proSP-B; CRP, C reactive protein; IL-6, interleukin 6; HSA-cys, cysteinylated albumin; DLCO, Lung diffusion for carbon dioxide; DLNO, Lung diffusion for nitric oxide; AU, arbitrary units. Data are expressed as median and interquartile range, median (Q1; Q3).

**Table 2:** General linear models generated considering HDL-SPB as dependent variable adjusting for age, gender, and HDL-C (Model 1 described in Methods section). In addition, model 1A considers cigarettes/day, model 1B pack-years, and model 1C smoking years as independent variable.

Parameter	Beta	95% Confidence Limits		*p*-Value
**Model 1A**				
Intercept	1.156	0.569	1.743	0.000
Gender	−0.087	−0.226	0.052	0.217
Age	0.010	−0.006	0.024	0.211
Cigarettes/day	0.032	0.021	0.043	**<001**
HDL-C	−0.006	−0.011	−0.001	**0.026**	
**Model 1B**				
Intercept	1.309	0.722	1.895	<0001
Gender	−0.093	−0.232	0.047	0.190
Age	0.005	−0.010	0.020	0.512
Log pack-years	0.454	0.300	0.609	**<001**
HDL-C	−0.006	−0.012	−0.001	**0.017**	
**Model 1C**				
Intercept	1.451	0.821	2.081	<0001	
Gender	−0.077	−0.225	0.071	0.303
Age	0.003	−0.014	0.019	0.758	
Smoking years	0.025	0.014	0.035	**<001**
HDL-C	−0.007	−0.013	−0.002	**0.011**

**Table 3:** General linear models generated considering HDL-SPB as dependent variable taking into consideration inflammatory and oxidative stress markers (Model 2 described in Methods section). In addition, model 2A considers cigarettes/day, model 2B pack-years, and model 2C smoking years as independent variable.

Parameter	Beta	95% Confidence Limits		*p*-Value
**Model 2A**				
Intercept	0.651	−0.208	1.510	0.135
Gender	−0.080	−0.230	0.071	0.296
Age	0.009	−0.007	0.024	0.263
Cigarettes/day	0.032	0.020	0.044	**<001**
HDL-C	−0.005	−0.011	0.001	0.093
Log WBC	0.415	−0.223	1.053	0.199
Log IL-6	−0.024	−0.312	0.264	0.868
Log CRP	−0.040	−0.163	0.084	0.524
HSA-cys	0.012	−0.016	0.040	0.403
**Model 2B**				
Intercept	0.765	−0.094	1.624	0.080
Gender	−0.084	−0.235	0.066	0.266
Age	0.004	−0.011	0.020	0.592
Log pack-year	0.450	0.289	0.611	**<001**
HDL-C	−0.006	−0.012	0.000	0.068
Log WBC	0.456	−0.177	1.088	0.156
Log IL-6	−0.045	−0.330	0.241	0.755
Log CRP	−0.038	−0.161	0.085	0.540
HSA-cys	0.012	−0.016	0.040	0.385
**Model 2C**				
Intercept	0.888	−0.037	1.814	0.060
Gender	−0.067	−0.227	0.093	0.405
Age	0.002	−0.015	0.019	0.795
Smoking years	0.024	0.013	0.035	**<001**
HDL-C	−0.007	−0.013	0.000	**0.038**
Log WBC	0.504	−0.171	1.178	0.141
Log IL-6	−0.108	−0.410	0.194	0.479
Log CRP	−0.015	−0.146	0.115	0.817
HSA-cys	0.011	−0.019	0.041	0.474

## Data Availability

Data collected in the study will be made available using the data repository Zenodo (https://zenodo.org/) with restricted access upon request to direzione.scientifica@ccfm.it.

## Supplementary Materials

The following are available online at https://www.mdpi.com/article/10.3390/biom11040551/s1, Table S1: Univariable Spearman correlations.
