# Supplementary material for: Immature Circulating SP-B, Bound to HDL, Represents an Early Sign of Smoke-Induced Pathophysiological Alterations
Source: Biomolecules. 2021 Apr 9;11(4):551. doi: 10.3390/biom11040551 (PMC8069080; doi:10.3390/biom11040551)
Supplement: Supplementary file 1 [file biomolecules-11-00551-s001.pdf]

Table S1· Univariable Spearman correlations.

Variable		HDL-SPB	Gender	Age	Cigarettes/day	smoking years	pack-years	HSA-cys	CRP	IL-6	WBC	DLCO	DLNO	HDL-C
**HDL-SPB**	*r value*	1·000	0·147	0·134	0·605#	0·544#	0·589#	0·007	-0·070	0·071	0·257§	0·017	0·104	-0·351#
**Gender**	*r value*	0·147	1·000	0·038	0·148	0·098	0·124	0·152	-0·150	0·064	-0·043	0·762#	0·803#	-0·515#
**Age**	*r value*	0·134	0·038	1·000	0·155	0·292§	0·248*	0·046	-0·147	0·036	0·075	-0·095	-0·095	-0·128
**Cigarettes/day**	*r value*	0·605#	0·148	0·155	1·000	0·912#	0·974#	-0·131	0·021	0·017	0·139	-0·092	0·048	-0·238*
**Smoking years**	*r value*	0·544#	0·098	0·292§	0·912#	1·000	0·972#	-0·115	0·003	0·046	0·132	-0·142	-0·028	-0·217*
**Pack-years**	*r value*	0·589#	0·124	0·248*	0·974#	0·972#	1·000	-0·104	0·016	0·028	0·141	-0·119	0·016	-0·236*
**HSA-cys**	*r value*	0·007	0·152	0·046	-0·131	-0·115	-0·104	1·000	0·102	0·048	0·163	0·196	0·190	0·027
**CRP**	*r value*	-0·070	-0·150	-0·147	0·021	0·003	0·016	0·102	1·000	0·449#	0·265*	-0·066	-0·131	0·116
**IL-6**	*r value*	0·071	0·064	0·036	0·017	0·046	0·028	0·048	0·449#	1·000	0·379#	0·034	-0·003	-0·309§
**WBC**	*r value*	0·257§	-0·043	0·075	0·139	0·132	0·141	0·163	0·265*	0·379#	1·000	-0·094	-0·101	-0·262*
**DLCO**	*r value*	0·017	0·762#	-0·095	-0·092	-0·142	-0·119	0·196	-0·066	0·034	-0·094	1·000	0·938#	-0·328§
**DLNO**	*r value*	0·104	0·803#	-0·095	0·048	-0·028	0·016	0·190	-0·131	-0·003	-0·101	0·938#	1·000	-0·395#
**HDL-C**	*r value*	-0·351#	-0·515#	-0·128	-0·238*	-0·217*	-0·236*	0·027	0·116	-0·309§	-0·262*	-0·328§	-0·395#	1·000

*=<0·05, §=<0·01 e #=<0·001
